# The application of custom 3D-printed prostheses with ultra-short stems in the reconstruction of bone defects: a single center analysis

**DOI:** 10.3389/fbioe.2024.1349819

**Published:** 2024-01-25

**Authors:** Peng Zhang, Wen Tian, Po Li, Fan Zhang, Guoxin Qu, Xinhui Du, Guancong Liu, Xiaoying Niu

**Affiliations:** Department of Orthopedic and Soft Tissue, The Affiliated Cancer Hospital of Zhengzhou University & Henan Cancer Hospital, Zhengzhou, China

**Keywords:** 3D-reprinting prostheses, ultra-short stem, malignant bone tumor, bone defect reconstruction, reconstruction

## Abstract

**Objective:** Considering the advantages and widespread presence of 3D-printing technology in surgical treatments, 3D-printed porous structure prostheses have been applied in a wide range of the treatments of bone tumor. In this research, we aimed to assess the application values of the 3D-printed custom prostheses with ultra-short stems for restoring bone defects and maintaining arthrosis in malignant bone tumors of lower extremities in children.

**Methods:** Seven cases of pediatric patients were included in this study. In all cases, the prostheses were porous titanium alloy with ultra-short stems. MSTS 93 (Musculoskeletal Tumor Society) scores were recorded for the functional recovery of the limbs. VAS (Visual analogue scale) scores were utilized to assess the degree of painfulness for the patients. X-ray and MRI (magnetic resonance imaging) were applied to evaluate the bone integration, prostheses aseptic loosening, prostheses fracture, wound healing, and tumor recurrence during follow-up.

**Results:** During follow-up, none of the patients developed any postoperative complications, including prostheses aseptic loosening, prostheses fracture, or tumor recurrence. Radiological examinations during the follow-up showed that prostheses implanted into the residual bone were stably fitted and bone defects were effectively reconstructed. The MSTS 93 scores were 24.9 ± 2.9 (20–28). VAS scores were decreased to 5.8 ± 1.2 (4.0–7.0). No statistically significant differences in leg length discrepancy were observed at the time of the last follow-up.

**Conclusion:** 3D-printing technology can be effectively applied throughout the entire surgical treatment procedures of malignant bone tumors, offering stable foundations for the initial stability of 3D-printed prostheses with ultra-short stems through preoperative design, intraoperative precision operation, and personalized prosthesis matching. With meticulous postoperative follow-up, close monitoring of postoperative complications was ensured. These favorable outcomes indicate that the utilization of 3D-printed custom prostheses with ultra-short stems is a viable alternative for reconstructing bone defects. However, further investigation is warranted to determine the long-term effectiveness of the 3D-printing technique.

## 1 Introduction

Bone tumors are tumors that occur in bone or originate from various components of bone tissues, among which malignant bone tumors account for about 40% (Niu et al., 2015). Malignant bone tumors, including osteosarcoma and Ewing sarcoma (Zeytoonjian et al., 2004), have higher incidence in children and adolescents (Muscolo et al., 2003), Malignant bone tumors are characterized by distal metastasis and the 5-year survival rates of patients are approximate 20% (Loh et al., 2014). The predilection sites of malignant bone tumors are commonly located in the long tubular bones of the extremities, such as the femur and tibia (Hmada et al., 2020). Abnormal and diffuse growth is a hallmark of malignant bone tumors, accompanied with a wide range of invasion, blurred boundaries with adjacent tissues, and high heterogeneity (Shin et al., 2000), which are prominent challenges for accurate and complete tumor resection and bone defect reconstruction in clinical treatment.

In recent years, the vigorous development of neoadjuvant chemoradiotherapy and accurate evaluation of preoperative imaging data are important breakthroughs in the treatment of malignant bone tumors, which has become the first choice for the limb salvage surgery (Mavrogenis et al., 2012; Jauregui et al., 2018). Limb salvage surgery mainly involves the complete resection of tumors and the effective reconstruction of bone defects. In traditional limb salvage surgery, the location, range, boundary, and surrounding tissue of tumor lesions are roughly evaluated by imaging data and treatment experience, with a higher risk of the incomplete or excessive resection of tumors, leading to increasing intraoperative bleeding (Ayvaz et al., 2014; Ozaki et al., 1996; O'Connor and Sim, 1989). At the same time, postoperative functional reconstruction is another great barrier in limb salvage surgery.

With the vigorous development of digital medical technology, 3D-printing technology has begun to emerge in the clinical treatment of malignant bone tumors. 3D-printing technology is fully integrated with preoperative imaging technology, in combination with a variety of new biomterials to accurately design a 3D digital model by the way of printing and stacking layer by layer, which has the characteristics of high compatibility and high biosecurity (Ma et al., 2018; Feng et al., 2021). However, few studies have reported on the clinical efficacy of 3D-printed prostheses in children with malignant bone tumors. Therefore, this study aims to retrospectively analyze malignant tumors in children with extreme osteotomy and preservation of the metaphysis, so as to provide a theoretical basis for 3D-printing technology in tumor resection and bone defects after resection.

## 2 Materials and methods

### 2.1 Patients

**FIGURE 1:**
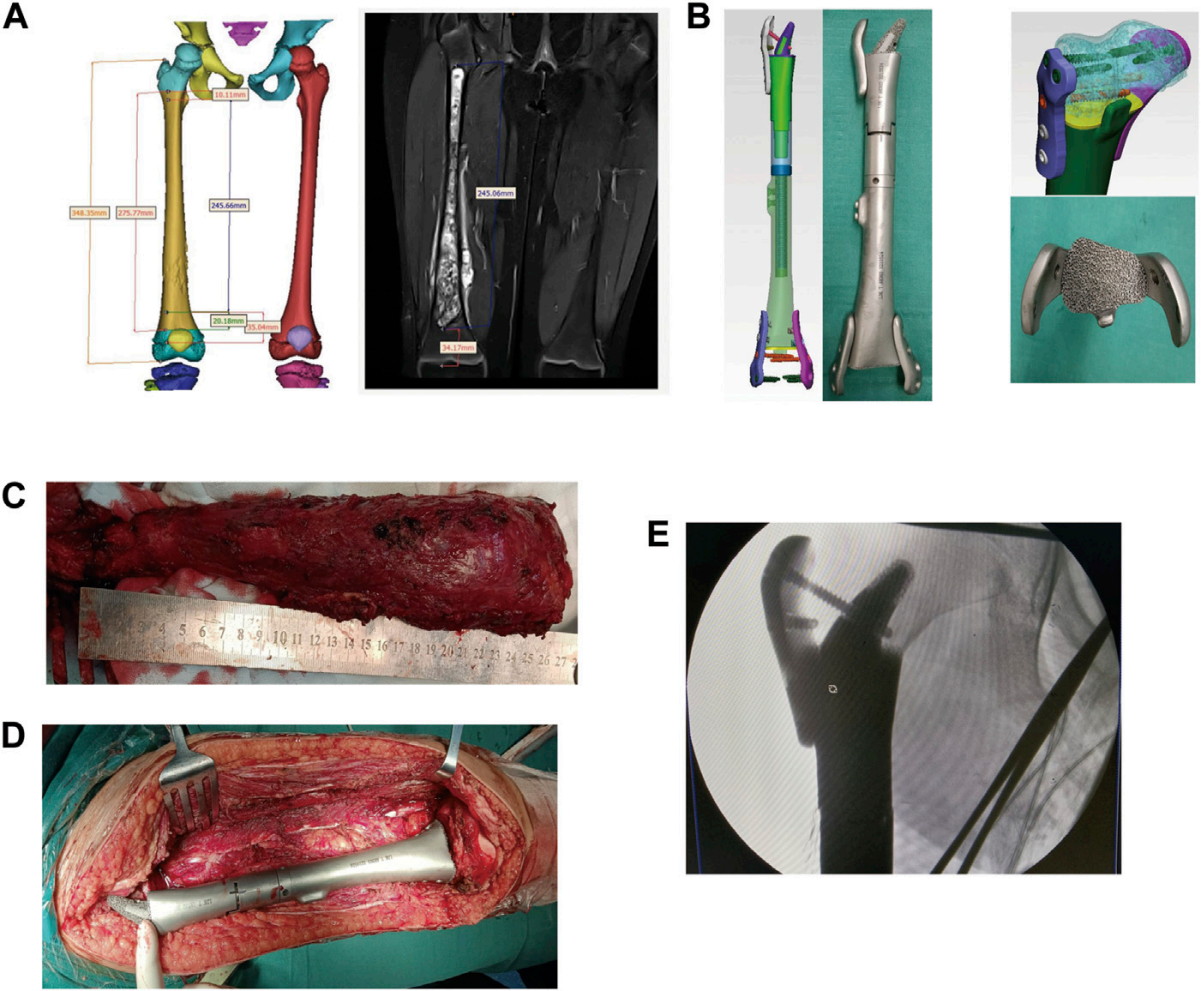
The reconstruction of the right tibia with a 3D-printed scaffold of a patient with osteosarcoma. **(A)** The design of the 3D-printed scaffold based on the MRI results. **(B)** The custom 3D-printed prosthesis components. **(C,D)**: The length measuring of the osteotomy. **(E)** The fixing of the custom 3D-printed prosthesis on the bone defect.

Among the seven cases, one was diagnosed with Ewing sarcoma and six were diagnosed with osteosarcoma. Patients with osteosarcoma were admitted to receive MAP + I (doxorubicin, cisplatin, high dose methotrexate, and ifosfamide) neoadjuvant chemotherapy. The one patient with Ewing sarcoma was referred to our hospital for a neoadjuvant chemotherapy regimen of VACIE (vincristine, doxorubicin, cyclophosphamide, ifosfamide, and etoposide) for 2 weeks, subjectively reduces pain in patients. After chemotherapy cycles of neoadjuvant chemotherapy, 3D-printed matching prosthesis was created for bone defect repair after tumor bone resection. Post-operative examination found no residual tumor (Figure 1).

### 2.2 Prosthesis design and manufacture

X-ray, computerized tomography, and MRI imaging examinations were performed after admission and preoperative chemotherapy.

Thin-slice computerized tomography was used as a data source, and MRI was used to determine the tumor scope and edema reaction area. After determining the lesion site and structural details, the original image was reconstructed in 3D and stored in DICOM format. The 3D reconstructed image was obtained by Simics software. Personalized 3D-printed prostheses for the patient were designed through the 3D design software UNIGRAPHICS NX. The porous structure of the prosthesis was designed using MAGICS software. After admission, computerized tomography, MRI, and X-ray examinations were performed before neoadjuvant chemotherapy and after chemotherapy, and the imaging data were compared before and after chemotherapy to determine the osteotomy location after excluding the edema area.

The lesions and surrounding soft tissues after resection of the tumor, the prosthesis was installed into the partial site and fit well with the surrounding tissue, to determine the invading of the tumor to the epiphyseal line, to evaluate the values of preserving the epiphyseal line and articular surface, and to plan the scope of the osteotomy. Finally, the distances were located 1.2 ± 0.6 cm (1.0–1.9 cm) away from the epiphyseal line. The prostheses were designed according to the local bone defect after surgical resection and the lengths of the contralateral femora. The components of the prostheses were both made of a titanium alloy porous structure.

**FIGURE 2:**
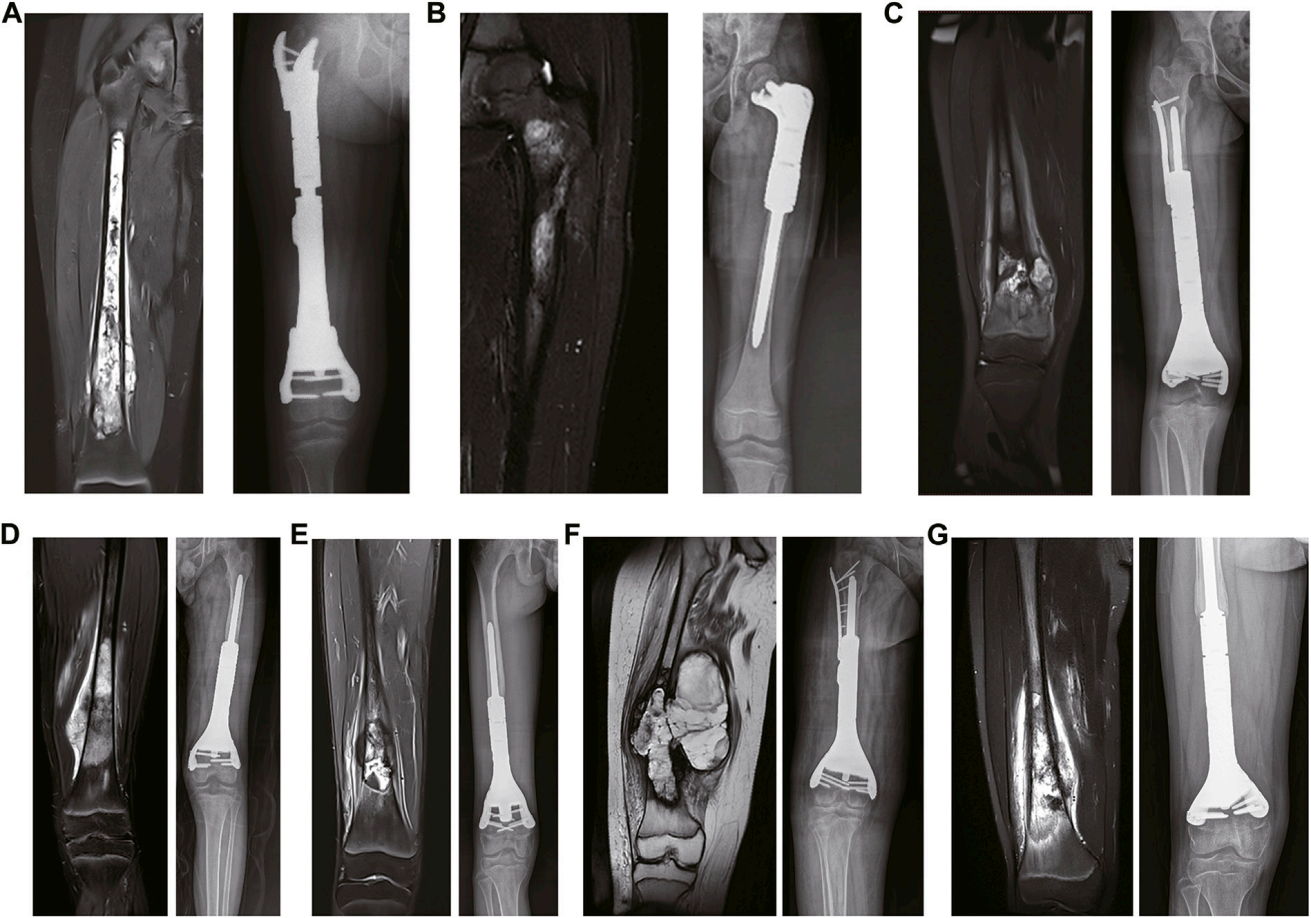
The preoperative and postoperative MRI results. **(A)** The preoperative coronal MRI results of case 1. **(B)** The preoperative coronal MRI results of case 2. **(C)** The preoperative coronal MRI results of case 3. **(D)** The preoperative coronal MRI results of case 4. **(E)** The preoperative coronal MRI results of case 5. **(F)** The preoperative coronal MRI results of case 6. **(G)** The preoperative coronal MRI results of case 7.

Next, one patient (case 1) with osteosarcoma was focused on. The proximal part of the prosthesis was designed as a horn shape due to the long lesion site. The osseous joint segment was designed to be porous with a porosity of 60%–70% and a pore diameter of 600 μm. The front and back components of the prosthesis were fixed with a short moment arm, combined with a plate or an interlocking nailing (Figures 2A, B).

**FIGURE 3:**
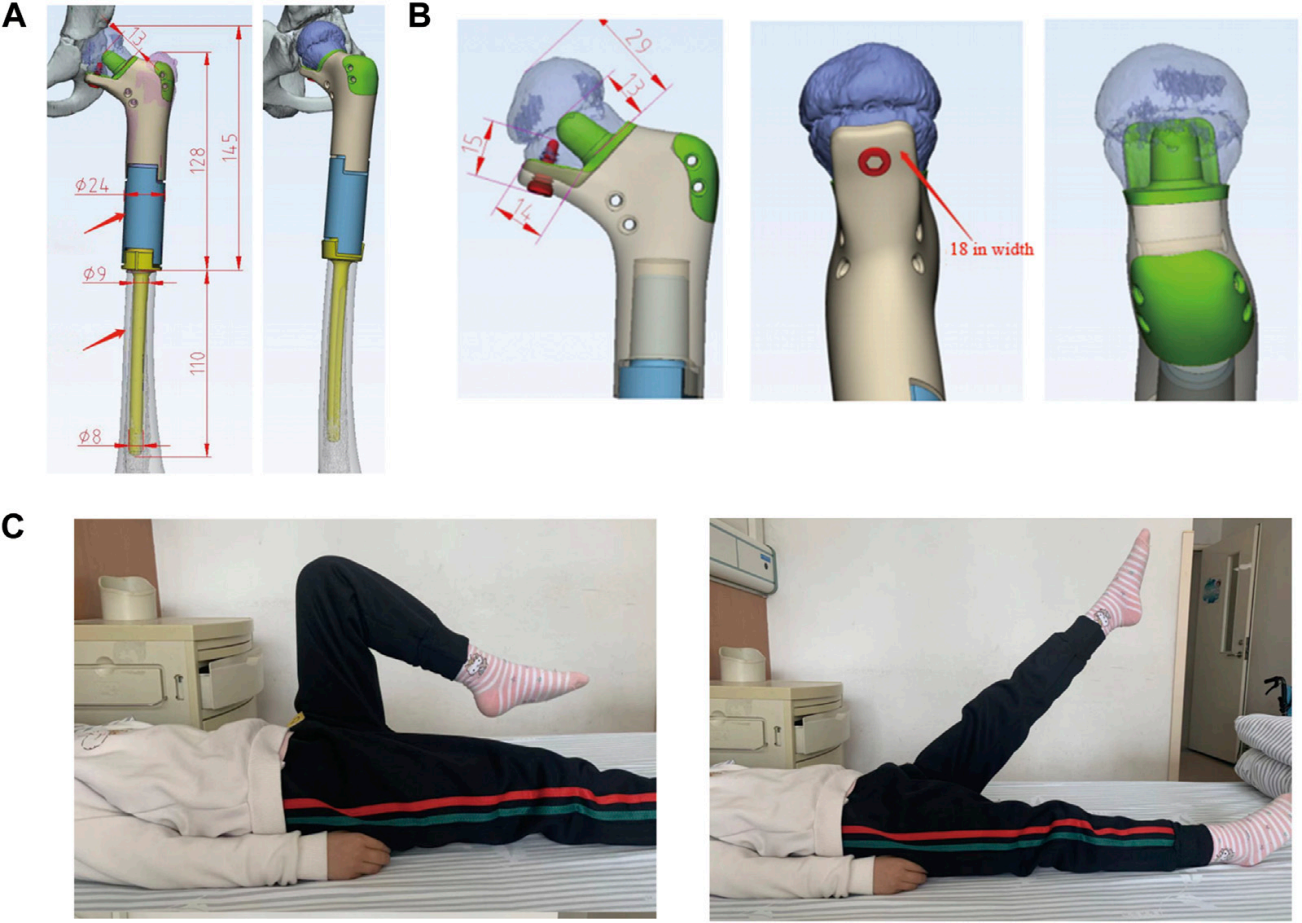
**(A)** 7-year-old female patient with Ewing sarcoma undergoing femur tumor resection and wedge osteotomy of the femoral neck and reconstruction with a 3D-printed scaffold. **(A, B)**: The extents of the personalized 3D-printed scaffold of the left proximal femur. **(C)**: Postoperative hip joint mobility at 6 months.

The prosthesis of the 7-year-old patient (case 2) with Ewing sarcoma consisted of the following components: (1) the proximal end of the prosthesis consisted of a “banana stalk” structure only 13 mm away from the epiphyseal line, with a diameter of 11mm–9mm; (2) the proximal prosthesis was supported by a plate with a width of 1.8 cm and a length of 1.4cm, and was fixed on the residual cortical; and (3) the distal end of the prosthesis consisted of a medullary needle (Figures 3A, B). Subsequently, the prostheses were well-preserved for operation after disinfection treatment.

### 2.3 Surgical procedures

All patients underwent general anesthesia with endotracheal intubation. In the supine position, the anterolateral femur was approached midline, with careful separation of the skin, subcutaneous tissue, muscle groups, blood vessels, and nerves layer by layer to fully expose the tumor truncated bone. Osteotomy was performed according to the preoperative 3D-printed osteotomy guide plate design, and the anatomical structure of the articular surface was preserved (Figures 2C, D, E, F). As listed in Table 1, the osteotomy length of the patients was 13.0–27.6 cm, respectively, with an average of 18.1 cm. Subsequently, the residual bone marrow cavity was reamed and the personalized 3D-printed prosthesis was inserted into the tibia to reconstruct the bone defect. A cancellous bone screw or the auxiliary plate screw was used to secure the implant and the residual bone. Finally, the surrounding muscle tissue was reconstructed to cover the prosthesis, the deep fascia and skin were sutured, and intravenous antibiotics were given to prevent postoperative infection.

**TABLE 1:** The detailed characteristics of patients.

Variables	Case 1	Case 2	Case 3	Case 4	Case 5	Case 6	Case 7
Age (year)	10	7	13	12	16	12	10
Gender	Female	Female	Female	Male	Male	Male	Female
Pathological diagnosis	Osteosarcoma	Ewing sarcoma	Osteosarcoma	Osteosarcoma	Osteosarcoma	Osteosarcoma	Osteosarcoma
Predilection sites	Right femur	Left femur	Right femur	Right femur	Left tibia	Right femur	Left tibia
Lesion length (cm)	24.0	7.6	14.5	11.1	17.3	13.6	11.2
Osteotomy length (cm)	27.6	13.0	17.5	17.6	20.0	16.6	14.2
Distance between epiphyseal line (cm)	1.5	1.3	1.5	1.0	1.7	1.9	1.0
Follow-up time (months)	1.6	1.0	1.1	0.8	1.1	1.6	1.0
Postoperative MSTS score	26	28	22	24	27	20	27
Growth in stature (cm)	5	4	7	6	7	5	7
Difference in length between two lower limbs (cm)	0	0	3	1	1	4	1
Preoperative VAS score	5	4	7	6	7	5	7
Postoperative VAS score	0	0	1	1	2	0	1

### 2.4 Postoperative management

*The patient continued the same chemotherapy regimen for 4 weeks after surgery*. The insertion statuses of the custom 3D-printed prostheses were examined postoperatively (Figure 2E). The affected limbs were pressurized by elastic bandages. After the recovering from anesthesia, functional recovery trainings of the patients were performed. The patients could walk with partial weight bearing on both crutches at 6 weeks postoperative and undertake full weight-bearing exercises at 3 months postoperative (Figure 3C).

### 2.5 Postoperative assessment

All patients were followed up regularly. X-ray and computerized tomography results of the affected limb were reviewed after operation. Additionally, prosthesis position, prosthesis function, and bone ingrowth were reviewed. MSTS score was recorded for the improvement of the functional recovery of the affected limb after operation, with a total of 30 points. A higher score indicated better recovery of limb function. VAS score was performed to assess the pain index of the patients with a total score of 10 points. A lower score showed a lower intensity of pain for patients.

## 3 Results

All patients were successfully operated on and followed up after operation, and the results revealed that patients were alive without postoperative complications, such as infection. No prosthesis loosening or periprosthetic fracture were observed. Neither tumor recurrence nor distant migration were found. At the same time, the wound and the function of limbs were well recovered after operation.

As one of the classic cases, one of the predilection sites was located in the right femur, of which the lesion length was 24.0 cm and the length of the intraoperative osteotomy was 27.5 cm with a distance of 1.5 cm from the epiphyseal line. Figure 2E shows the stably fixed prosthesis and the equality in length of the lower limbs at 3 months postoperative. The results of postoperative follow-up at 6 months showed that the MSTS score was 26, which is graded as excellent. At a follow-up at 16 months, the height of patients increased by 8 cm and the length differences between the limbs was 1 cm. The VAS score was 5 before surgery and 0 after surgery, and there was significantly improved compared with the preoperative VAS score. Videos taken during the 3-month and 6-month follow-up are shown in Supplementary Videos S1, S2, respectively.

The predilection site of the patient with Ewing sarcoma was located in the proximal left femur, with a lesion length of 7.6 cm and an intraoperative osteotomy of 13.0 cm. The distance between the distal osteotomy and epiphyseal line was 1.3 cm. No prosthetic loosening and fracture were found and bone ingrowth was in good condition during the 12-month follow-up. After following up for 12 months, the MSTS score was 28, the increase in height was 5.0 cm and no length differences between the operative limb and the healthy limb were observed. Compared with the preoperative VAS score of 4, the postoperative VAS score was significantly reduced to 0. The details of all patients are listed in Table 1. The follow-up videos of 3 months and 6 months postoperative are shown as Supplementary Videos S3, S4, respectively.

## 4 Discussion

In this study, titanium alloy as the surface material and the porous structure of bone trabeculae were selected to design and synthesize personalized 3D-printed prostheses for the bone defect reconstruction of two pediatric patients the reason for the choice of prosthetic reconstruction was the resection of the tumor. After continuous follow-up, the prostheses were stably integrated with the residual bone, and fracture, loosening, or infection around the prosthesis were not observed. The good recovery of limb functions was beneficial for patients to return to normal life. In the past, there was no effective reconstruction method for this ultra-short femoral osteotomy, and only half hip arthroplasty or total hip arthroplasty could be performed. Postoperatively, the children had a serious problem of unequal length of lower limbs. The results of our center studies (Zhang et al., 2023) including this research indicated that 3D-printing technology could provide a safe and effective niche for bone reconstruction in the clinical treatment of pediatric malignant bone tumors.

The application of 3D-printed personalized models in bone tumor surgery can effectively assist tumor resection. The complex anatomical structures of the tumor site can be transmitted into a 3D digital model with the combination of 3D-printing technology and preoperative computerized tomography and MRI data according to the clinical needs, which can reflect the tumor margin and the relationship of the tumor to the vital structures, including the vessels and nerves (Rengier et al., 2010; Duncan et al., 2015). Compared with traditional tumor resection depending on experience, 3D-printed models have the characteristics of allowing surgeons to make more accurate judgments when planning the surgical procedure and protecting the main blood vessels and nerves, which effectively avoids non-essential amputation or R1 resection (Xiao et al., 2016; Kwakwa et al., 2017; Van Genechten et al., 2021). The 3D-printed model could transmit the heterogeneous tumor environment information of various patients to surgeons to provide guidance for preoperative planning, thereby leading to a solid foundation for total radical tumor resection (Ozturk et al., 2019). In addition, 3D-printed models are designed for preoperative planning, resulting in the significant decrease of surgical and fluoroscopy times without preoperative simulation of 3D printed prosthesis models, clinicians cannot accurately and quickly install prostheses by experience (Cherkasskiy et al., 2017). In this study, the preoperative imaging data were bonded with 3D-printing technology to make customized 3D-printed models of the patients, which were conducive to accurately possess the structures of tumor areas and surrounding tissues and to discuss the tumor excision, of which the osteotomies were performed at a distance of 1–3 cm from the tumor boundary. Postoperative results showed that the tumors were completely removed and the epiphyseal lines were preserved to retain the growth potential of the affected limbs in the future. In addition, the personalized models were presented to the patients to roughly understand the operative programs beyond the obscure and professional fields of medicine, in order to reduce the fear of surgery in children.

Remodeling large bone defects after tumor resection is another great challenge in the clinical treatment. Previous studies have reported multiple methods of bone defect reconstruction, such as autologous tumor bone inactivation (Baldwin et al., 2019), allogeneic bone transfer (Zavras et al., 2022), and Ilizarov distraction osteogenesis (Demiralp et al., 2014). However, due to tumor recurrence, delayed wound healing, bone non-union, and infection (Grimer, 2005), the short-term effects of these biological reconstruction methods are poor. In our study, 3D-printing technology was selected to complete the prostheses synthesis of the two patients using titanium alloy materials. The major components of titanium alloy are Ti6AI4V, which is similar to the elastic modulus of bone (Arabnejad et al., 2017), of which the elastic modulus of cortical bone and cancellous bone are 0.5 and 10–20 GPa, respectively (Zhang et al., 2017). Considering the advantages of biological similarity and biocompatibility, prostheses made from titanium alloy materials are more suitable to the application of bone reprogramming. A 12-year-old patient with Ewing sarcoma underwent the rebuild of a 3D-printed prosthesis, which was made of titanium alloy material. Postoperative follow-up results showed that the prosthesis was stably integrated with residual bone, showing good biomechanical stability (Xu et al., 2016). Consistent with the above study, the postoperative follow-up results of pediatric patients in our center with 3D-printed titanium alloy prostheses showed that no fracture or loosening were found, which was beneficial to the long-term stability.

The gradual fusion of 3D-printed titanium alloy prostheses (Geng et al., 2021; Meng et al., 2022) and residual bone surface mainly depends on bone ingrowth. Bone ingrowth refers to the process in which osteoblasts secrete and synthesize bone collagen and bone protein fibers at the target site, accompanied with precipitate calcium and phosphorus in the pores of the prostheses, in order to provide a potential breeding ground for the generation of bone tissue and finally generate new bone tissue, which is an effective index to evaluate the long-term combination of the prostheses and bones (Pauly et al., 2012; Rentsch et al., 2014; Gu et al., 2018). Previous studies have shown that the pore structure of prosthesis materials could significantly optimize the histocompatibility between prostheses and residual bone to harbor cell adhesion and proliferation to promote bone ingrowth. Studies have revealed that compared with the (15.0 ± 2.9)% and (37.9 ± 4.0)% pore size groups, the viability of MSCs (mesenchymal stem cells) were highest in the (15.0 ± 2.9)% pore size titanium alloy prostheses (Cheng et al., 2014). Similarly, BMSCs (bone marrow stromal cells) were seeded on titanium alloy porous scaffolds with porosities of 40% and 70%, respectively. The results showed that a 70% pore size was more conductive to the adhesion and proliferation of cells (Wang and Li, 2016). Not only the porosity of the prosthesis but also the pore size affects bone ingrowth. Studies have evaluated the fixation abilities of titanium alloy prostheses with diameters of 300, 600, and 900 μm after implantation, of which the results showed that 600 μm proved a niche for implants due to the traits of the appropriate strength and high holding ability (Taniguchi et al., 2016). Another study reported that compared with the 1,200 um pore size scaffold, the cells implanted in the 600 um pore size scaffold acquired the characteristics of stronger active viability, alkaline phosphatase activities, and bone mineralization abilities (Lv et al., 2015). In this study, the relationships between porosity, pore size, and the mechanical strength of titanium alloy plates and elastic modulus were comprehensively considered. Finally, the titanium alloy with a porous structure of 60%–70% porosity and 600um pore size was selected, with an auxiliary plate and interlocking screw to enhance the stability of the prosthesis and bone interface. The results of postoperative follow-up found that the prostheses and screws of two patients were not loose and were stably integrated with the bones, suggesting that a porous titanium alloy bracket with 60%–70% porosity and 600um pore size could meet the requirements of the physiological and mechanical properties of daily life and have reliable application prospects in the future.

For patients with malignant bone tumors, the reconstruction of bone defects after resection of bone tumors has always been an enormous challenge for surgeons (Chen et al., 2022). The primary purpose of prosthetic construction after femoral tumor resection is to effectively restore lower limb function to help patients back to a normal life. In recent years, with the exponential development of 3D-printing technology and in-depth analysis of osseointegration theory, the 3D-printed long-backbone prosthesis has been an emerging application for the hallmarks of good stability and the personalized matching of bone defect sites. In this study, the two patients are children who needed to retain osteoepiphyses and joint planes on the basis of complete resection of tumor segment bone to maintain normal physical growth ability and avoid the phenomenon of double lower limbs. Subsequently, the osteotomy was performed at a distance of 1–3 cm from the epiphyseal line, where the porous structural prostheses were placed at the end of the osteosynthesis. Due to withstanding no compressive force and lateral stress, the scaffolds were steadily fused with the residual bone to achieve the purpose of long-term stability. Furthermore, the traditional mechanical processing procedures were adopted for the ultra-long prostheses to avoid excessive stress concentration to effectively reduce the absorption of the proximal femoral bone while ensuring mechanical stability, which finally achieved good effects in the early and middle periods of the treatments.

However, our study still has some limitations. Firstly, this was a retrospective study with a small sample size. As the number of cases was only two, it was difficult to analyze the relationship between the scope of surgical resection and disease progression. Secondly, the relevant finite element stress analyses of the prostheses were not performed, thus the stress situations of the prostheses could not be clearly clarified. Thirdly, the longest follow-up time for the patients in this study was 9 months. Although the initial follow-up results were good, the correlations between long-term 3D-printed prosthetic bone defects and postoperative limb function recovery, survival status, and quality of life in patients need to be further proved.

## 5 Conclusion

3D-printed titanium alloy porous structure prostheses with ultra-short shanks can fully preserve the physiological function of adjacent articular surfaces to the limit, and screw fixation contributes to the stability of the prostheses stalk. Through the preoperative simulation of 3D-printed models, the intraoperative assisted resection of the tumors and personalized matching of bone defect sites, and paying more attention to postoperative rehabilitation trainings, the recovery of limb function of patients can be accelerated. However, longer follow-ups and a larger sample size are still needed to further prove the application reliability of 3D-printed titanium alloy porous structure prostheses with ultra-short shanks.

## Data Availability

The original contributions presented in the study are included in the article/Supplementary Material, further inquiries can be directed to the corresponding author.
